# Utility of a Short Neuropsychological Protocol for Detecting HIV-Associated Neurocognitive Disorders in Patients with Asymptomatic HIV-1 Infection

**DOI:** 10.3390/brainsci11081037

**Published:** 2021-08-04

**Authors:** Martha Martinez-Banfi, Jorge I. Vélez, Moisés R. Mebarak Chams, Mauricio Arcos-Holzinger, Johan E. Acosta-López, Ricardo García, María Victoria Perea, Mauricio Arcos-Burgos, Valentina Ladera

**Affiliations:** 1 Facultad de Ciencias Jurídicas y Sociales, Universidad Simón Bolívar, Barranquilla 080005, Colombia; jacosta@unisimonbolivar.edu.co; 2 Department of Industrial Engineering, Universidad del Norte, Barranquilla 081007, Colombia; jvelezv@uninorte.edu.co; 3 Department of Psychology, Universidad del Norte, Barranquilla 081007, Colombia; mmebarak@uninorte.edu.co; 4 Grupo de Investigación en Psiquiatría (GIPSI), Departamento de Psiquiatría, Instituto de Investigaciones Médicas, Facultad de Medicina, Universidad de Antioquia, Medellín 050010, Colombia; oscararcos98@gmail.com (M.A.-H.); mauricio.arcos@udea.edu.co (M.A.-B.); 5 Facultad de Psicología, Universidad de Salamanca, 37008 Salamanca, Spain; rigar@usal.es (R.G.); vperea@usal.es (M.V.P.); ladera@usal.es (V.L.)

**Keywords:** HIV, AIDS, HAND, neurocognitive disorder, predictive models, neuropsychological screening, machine learning

## Abstract

Human Immunodeficiency Virus type 1 (HIV-1) infection is a chronic disease that affects ~40 million people worldwide. HIV-associated neurocognitive disorders (HAND) are common in individuals with HIV-1 Infection, and represent a recent public health problem. Here we evaluate the performance of a recently proposed short protocol for detecting HAND by studying 60 individuals with HIV-1-Infection and 60 seronegative controls from a Caribbean community in Barranquilla, Colombia. The short evaluation protocol used significant neuropsychological tests from a previous study of asymptomatic HIV-1 infected patients and a group of seronegative controls. Brief screening instruments, i.e., the Mini-mental State Examination (MMSE) and the International HIV Dementia Scale (IHDS), were also applied. Using machine-learning techniques, we derived predictive models of HAND status, and evaluated their performance with the ROC curves. The proposed short protocol performs exceptionally well yielding sensitivity, specificity, and overall prediction values >90%, and better predictive capacity than that of the MMSE and IHDS. Community-specific cut-off values for HAND diagnosis, based on the MMSE and IHDS, make this protocol suitable for HAND screening in individuals from this Caribbean community. This study shows the effectivity of a recently proposed short protocol to detect HAND in individuals with asymptomatic HIV-1-Infection. The application of community-specific cut-off values for HAND diagnosis in the clinical setting may improve HAND screening accuracy and facilitate patients’ treatment and follow-up. Further studies are needed to assess the performance of this protocol in other Latin American populations.

## 1. Introduction

Human Immunodeficiency Virus type 1 (HIV-1) infection is a chronic disease [1] that affects 37.9 million people worldwide [2]. HIV-associated neurocognitive disorders (HAND) are common in individuals with HIV-1 Infection [3,4], and represent a recent public health problem [5]. HAND is a neurocognitive impairment of attention, concentration, and memory domains, along with motor signs [6] that affect a patient’s daily life. The HAND clinical profile follows the Frascati diagnostic criteria [7], and according to its severity, is categorized as Asymptomatic Neurocognitive Impairment (ANI), Mild Neurocognitive Disorder (MND), and HIV-Associated Dementia (HAD) [7]. Since the appearance of combined antiretroviral therapy (cART), HAD cases have declined (i.e., occurs in less than 5% of individuals undergoing cART), but MND remains frequent [3,8,9,10].

The neuropsychological evaluation represents a critical component of HAND diagnosis [10,11]. Several screening instruments and neuropsychological protocols are documented by the scientific literature, namely those proposed by the National Institute of Mental Health [12], the World Health Organization [13], the MACS [14], the CCWMS [15] and the HUMANS batteries [16]. More recently, batteries such as the Western Neuropsychological Test Battery of the HIV Neurobehavioral Research Center [11], and a battery developed by Brazilian researchers [17] are available. Although these comprehensive batteries are more sensible than screening tests [11,18], the lack of access to them in environments with limited resources [19] increases the need to develop screening tools and short assessment protocols that facilitate HAND detection [20,21]. Some of these screening instruments include the Mini-mental State Examination (MMSE) (scrutinized for its low sensitivity) [8,9], the HIV Dementia Scale (HDS) [22], and the International HIV Dementia Scale (IHDS) [23] (with limited performance especially for the diagnosis of MND) [18,24,25], the Cognitive Assessment Tool-Rapid version (CAT-rapid) (with borderline sensitivity and low specificity) [8], the Montreal Cognitive Assessment (MOCA, with acceptable sensitivity but low specificity) [8], and the CogState computerized battery (with a sensitivity of 76% and specificity of 71%, when compared to full neuropsychological testing) [21]. More recently, combinations of traditional [26] and short batteries of standard neuropsychological tests, which offer one of the best options to monitor the cognitive health of HIV+ individuals in clinics or research environments [27], have also been proposed as screening tests.

Despite this, a global consensus about which screening tools are most reliable is yet to be reached. This is partly due to specific differences in the cultural, economic, and linguistic training of evaluators, among other community aspects. Diagnostic tools should ideally have population-specific normative standards and evidence of cross-cultural validity for diagnostic reliability [28]. Additionally, applying full neuropsychological assessment would be ideal; however, in countries with less economic resources, these evaluations may not be available because of the cost and time required [29,30].

Good clinical practice suggests that early screening and follow-up of HAND with standardized tools [24] should be the routine assessment strategy for the cognitive performance of HIV patients [31]. Close monitoring of these patients would improve pharmacological treatments [18] and facilitate cognitive and behavioral interventions designed to improve the quality of life in HIV patients [5,17].

Clinical detection and HAND scrutiny with neuropsychological protocols can nurture new methods for prediction [20,32], which have recently been used in neuropsychological studies and have proven to be useful diagnostic tools in the clinical setting [33,34,35]. Among predictive models, machine learning (ML) algorithms showed an efficient performance to define HAND-specific treatment for individuals with advanced HIV [36,37]. One such algorithm is the Advanced Recursive Partitioning Approach (ARPA), widely used to build predictive tree-based ML models with Classification and Regression Tree (CART) analysis [38].

In this study, using ARPA, we assess the efficiency of a short version of a full neuropsychological protocol for HAND detection, recently proposed by our group [39]. We then compare it with other clinical screening instruments to define community-specific cut-off values for HAND diagnosis in individuals with asymptomatic HIV-1 Infection ascertained from a Caribbean community inhabiting Barranquilla, Colombia.

## 2. Subjects and Methods

### 2.1. Subjects

We recruited and clinically evaluated 60 individuals with asymptomatic HIV-1 Infection and an equal number of seronegative controls. These individuals correspond to those involved in a previous study by our group [39]. The inclusion criteria for individuals with HIV-1 Infection were (i) a diagnosis of HIV-1 Infection in the asymptomatic stage; (ii) age ranging between 18 and 58 y/o (to control any potential age-related cognitive immaturity or decline) [24]; (iii) the completion of at least two years of elementary school; (iv) a maximum time since HIV diagnosis of 9 years (considering nine years is the maximum period HIV-1 infected individuals can remain asymptomatic) [40]; and (v) no history of alcohol and/or drug use, neurological, neuropsychological, and/or psychopathological disorders before HIV-1 diagnosis. The target population is primarily of low socioeconomic status, making it more vulnerable to develop HAND [19]. On the other hand, the control group accomplished the same criteria but was not infected with HIV. Table 1 shows the demographic characteristics of all individuals enrolled in this study. The mean time since diagnosis in the HIV-1 group was 3.32 ± 2.5 months, and 81.67% of the patients were on cART.

### 2.2. Short Neuropsychological Protocol for HAND Detection

In a previous study by our group [39], a comprehensive neuropsychological gold standard battery was applied [7] to a group of individuals with asymptomatic HIV-1 Infection and a group of seronegative controls. Tests showing statistically significant differences between these groups were included in the short protocol considered in this study for HAND detection (Table 2). This protocol, described in detail in the Supplementary Material, includes neuropsychological tests used in similar studies in the U.S. [41], Germany [3], and Zambia [11], and categorized as ‘useful’ tools for HAND diagnosis [6], takes ~1 h to be applied. Furthermore, it also includes a psychological test for anxiety, and it is essential to clarify that as part of the comprehensive neuropsychological battery previously applied in our sample [39], we included the evaluation of activities of daily living. However, no statistically significant difference was found between individuals with asymptomatic HIV-1 Infection and seronegative controls. Hence, this test was not included as part of the short neuropsychological protocol presented herein. Despite this, it is highly recommended to include it as part of the clinical examination.

The standardized mental status examinations (MSEs) can be used with the appropriate demographically standardized cut-offs to evaluate HAND [7] during the early stages of the Infection [24], when full neuropsychological testing is not available. It is here where brief screening instruments, such as the Mini-mental State Examination (MMSE) [42] and the International HIV dementia scale (IHDS) [23], are widely used for HAND screening, especially in situations where there are time and resource limitations [5]. Although these are not part of our short protocol, these tests were also administered to all patients. Following standard guidelines, individuals scoring ≤26 points in the MMSE were considered to have mild cognitive impairment [43], and those with an IHDS score of ≤10 were subsequently evaluated for possible dementia [23].

To assess the HAND detection performance of our short neuropsychological protocol, and to compare it with the MMSE and IHDS screening tests, we defined HAND status according to three operational criteria: (1) MMSE ≤ 26 [44], (2) IHDS ≤ 10 [23], and (3) both MMSE ≤ 26 and IHDS ≤ 10—the latter determines whether the screening of cognitive impairment associated with both cortical and subcortical is present in HIV-1 infected individuals. Besides, we used the one-standard-deviation (1SD) criterion to assess suspicion of HAND in all patients [45]. This criterion translates into acquired impairment in cognitive functioning, involving at least two ability domains, based on age and education, and adjusted neuropsychological tests [45].

**Table 2:** Neurocognitive and psychological tests included in our proposed brief screening protocol for detecting HAND.

Neurocognitive and Psychological Domain	Test	Conventions
Attention span	Wechsler Memory Scale Digit Span Subtest [46]	T1 = Total score T2 = Direct Digit score
Verbal learning and memory	Rey Auditory Verbal Learning Test [47,48]	T5 = Trial 3 T6 = Trial 5 T7 = Total score in trials 1 to 5
Language *Phonemic verbal fluency*	Controlled Word Association Test [49]	T12 = Total score T13 = Total score in letter “P” T14 = Total score in letter “M”
*Vocabulary*	Wechsler Intelligence Scale Vocabulary Subtest [50]	T3
*Naming*	Boston Naming Test [51]	T9 = Phonemic clues
Auditory-verbal comprehension	Language Comprehension Subtest of the Brief Neuropsychological Assessment in Spanish [52]	T19
Information processing speed	Digit Symbol-Coding Subtest of the Wechsler Intelligence Scale (WAIS-III) [50]	T4
Visuoconstructive skills	Rey Complex Figure Test [53]	T8 = Total direct score copy
Executive functions *Inhibition*	Stroop Color-Word Test [54]	T10 = Total score (word) T11 = Total score (color)
*Motor programming*	Motor Skills Subtest of the Brief Neuropsychological Assessment in Spanish (NEUROPSI) [52]	T15 = Score in change of hand position T16 = Score in alternating movements of the two hands T17 = Score in opposite reactions T18 = Total score
Anxiety	State-Trait Anxiety Inventory (STAI) [55]	T20 = STAI (State) T21 = STAI (Trait)

### 2.3. Procedure

Full neuropsychological tests were completed in two sessions and did not represent any risk for the participants since they only included pen-and-paper-based tests routinely used in the clinical practice. Informed written consent was obtained from all participants [39]. This study was carried out following local review board approval.

### 2.4. Statistical Analysis

All the data in this study were processed and analyzed using R version 3.5.0 [56]. Means, standard deviations (S.D.s), and range measures were used to summarize continuous variables such as age. Frequency and proportions were used to describe categorical variables such as gender and sexual orientation. We used the Advanced Recursive Partitioning Approach (ARPA) to construct a predictive tree-based ML model of HAND status using Classification and Regression Tree (CART) analysis [38] as implemented in the “rpart” package [57] for R. ARPA is widely used in predictive analyses as it accounts for (i) non-linear hidden interactions better than other alternative methods, (ii) it is independent of the type of data and of the data distribution type, (iii) it offers fast solutions to reveal hidden complex substructures, and (iv) provides non-biased statistical analyses of high-dimensional, seemingly unrelated data [58]. In our analysis, gender, age, years of education, HIV-1 infection status (0 = seronegative control; 1 = HIV-infected), and variables collected in the neuropsychological battery were the predictors. A 5-fold cross-validation procedure was used to evaluate our ARPA-based predictive model of HAND for unobserved data. The performance of CART was assessed using the Receiver Operating Characteristic (ROC) curve [59] and the area under the ROC curve (AUC). The sensitivity (*S_e_*), specificity (*S_p_*), correct classification rate (CCR), positive predictive value (PPV), negative predictive value (NPV), false discovery rate (FDR), false-positive rate (FPR) and lift [60,61] were used as additional criteria (Supplementary Tables S1 and S2).

We implemented an iterative procedure to derive community-specific cut-off values for the MMSE and IHDS for this population based on the ARPA-based predictive model for HAND. For illustration purposes, suppose we are interested in deriving the optimal cut-off for the MMSE. The procedure begins by partitioning the MMSE range in a sequence of *k* equally spaced numbers such the sequence *x*_1_ < *x*_2_ < *x*_3_ <⋅⋅⋅< *x_k_* is obtained, where *x*_1_ and *x_k_* are, respectively, the minimum and maximum MMSE values in our cohort. Next, we define a “positive HAND screening” when MMSE < *x_j_*, where *x_j_* is a particular value of the aforementioned sequence. The *S_e_*, *S_p_*, and CCR performance measures for the ARPA-based prediction model were further estimated using this diagnosis. The optimal cut-off value for the MMSE in this cohort, e.g., *x*^+^, was defined as that maximum of both *S_e_* and *S_p_*. A similar procedure was used to establish the optimal cut-off value for the IHDS.

## 3. Results

### 3.1. Predictive Models for HAND Detection

#### 3.1.1. HAND Detection Based on the 1SD Criterion

**Figure 1:**
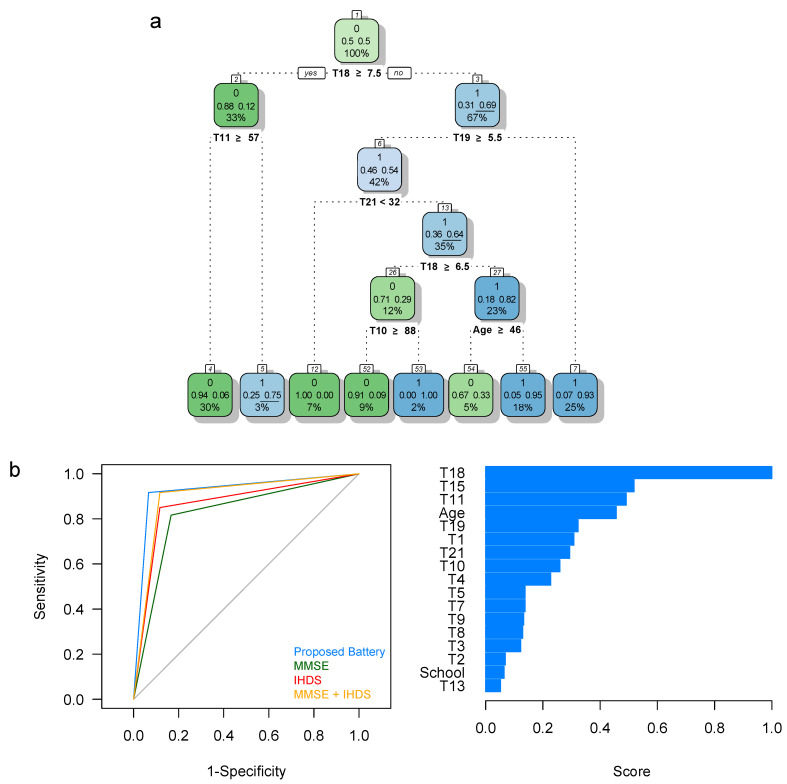
Results of the ARPA-based predictive model for HAND screening. (**a**) Classification tree derived using the CART strategy. HAND status was defined using 1 S.D. below the normative data and predicted using the proposed neuropsychological battery. Numbers within white squares represent the node number; the first line corresponds to the most frequent class (0: Unaffected; 1: HAND affected), the second line to the probability of each class within the node, and the third line to the percentage of the total sample size (*n* = 120) within each node. Nodes where HAND-affected individuals are more likely to be classified are shown in blue. (**b**) (**left**) ROC curves when HAND status was predicted using the ARPA-based model, including the proposed battery, MMSE, IHDS, and both MMSE and IHDS. (**right**) Variable importance for the derived predictive model based on the proposed battery. ARPA: Advanced recursive partitioning approach; CART: Classification and regression tree; ROC: Receiver operating characteristic. School: Years of education. Here, T1, T2,…, T21 correspond to the neuropsychological and psychological tests. See Table 2 for more details.

Sixty individuals met the 1SD criterion for HAND (Table 3). We derived a four-level classification tree to predict HAND status (Figure 1a). This HAND ARPA-based model yielded *S_e_*, *S_p_*, CCR, and AUC values above 0.9 for the full sample (Table 4), and includes the individual’s age, the T10 (Total Score in the Word component of the Stroop’s Color-Word Test), the T11 (Total Score in the Color component of the Stroop’s Color-Word Test), the T18 (Total Score in the Motor Skills subtests of the NEUROPSI), the T19 (Score in the Language Comprehension Subtest of the NEUROPSI), and the T21 (State-Trait Anxiety Inventory [Trait]) tests as predictors (Figure 1a). Thus, individuals with scores T18 < 7.5 have a 69% chance of HAND affection (that is, 67% of our sample; node 3). This figure reduces to 54% when, in addition, individuals score 5.5 or more in T19 (that is, 42% of our sample; node 6), and dramatically increases to 93% when individuals score less than 5.5 in this latter test (that is, 25% of our sample; node 7). Interestingly, individuals 46 y/o or younger, scoring at least 7.5 points in the T18, at least 5.5 points in the T19, more than 32 points in the T21, and less than 6.5 points in the T18, have a 95% chance of HAND affection (18% of our sample) (Figure 1a and Table 3). Comparison of the ROC curves (Figure 1b, left) and performance measures (Table 4) show that our proposed short protocol outperforms other HAND-detection neuropsychological instruments in our sample; the T18, T15, and T11 tests seem to be the most important variables for HAND diagnosis (Figure 1b, right).

#### 3.1.2. HAND Detection Based on the MMSE and IHDS Operational Criteria

Based on the MMSE criterion, we found no seronegative controls with suspicion of HAND, and a total of nine individuals with HIV-1 Infection were identified with suspicion of HAND (Table 5). The average MMSE differed between groups (HIV: 27.55 ± 1.9, control: 29.05 ± 1.11; *p* = 0.002).

**Figure 2:**
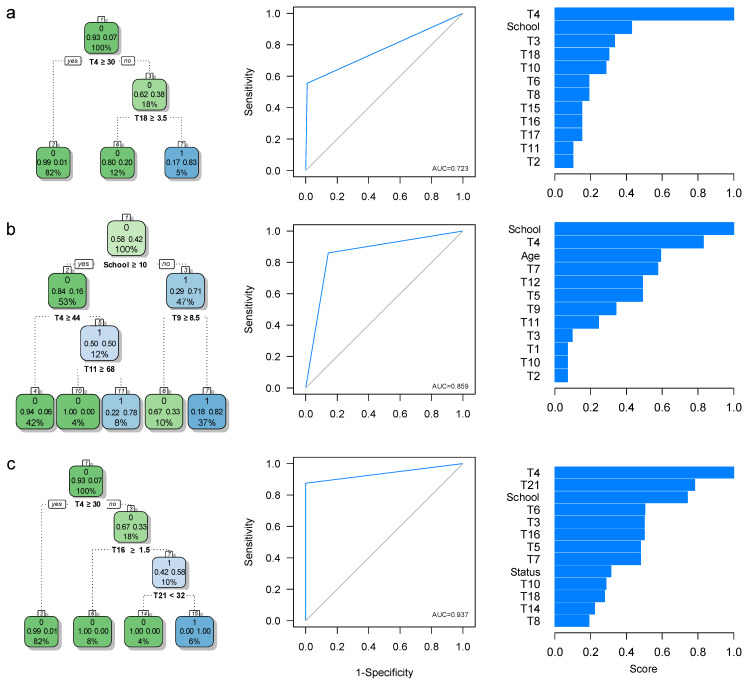
Results of the ARPA-based predictive model for HAND screening in individuals with HIV-1 Infection from the Colombian Caribbean. The HAND status was defined using (**a**) MMSE < 26, (**b**) IHDS ≤ 10, and (**c**) both MMSE < 26 and IHDS ≤ 10 (see Methods). (**left**) The classification tree derived using the CART strategy. Status: HIV-1 Infection status (0: control; 1: case). Other conventions as in Figure 1.

A two-node classification tree to predict HAND affection status was derived (Figure 2a). This tree is composed of the T4 (Digit Symbol-Coding Subtest of the Wechsler Intelligence Scale) and the T18 (Total Score in the Motor Skills subtests of the NEUROPSI) neuropsychological tests. Thus, individuals with scores T4 < 30 and T18 < 3.5 have an 83% chance of HAND diagnosis when the MMSE criterion is used (that is, 5% of our sample), and it correctly classifies 5/9 (55.5%) individuals initially identified as suspected of HAND, based on the MMSE criterion, leading to *S_e_* = 0.55, *S_p_* = 0.99, CCR = 0.958, and AUC = 0.723 (Figure 2a and Table 5).

Using the IHDS criterion, 50 individuals met the criteria for HAND (36 with HIV-1 Infection and 14 seronegative controls) (Table 5). The mean IHDS differed between the two assessed groups (HIV: 9.79 ± 1.97, control: 11.02 ± 1.21; *p* = 0.001). Our ARPA-based predictive model corresponds to a three-level tree and includes the years of education, the T4, the T11 (total score in the Color Component of the Stroop’s color-word test), and the T19 (score in the Language Comprehension Subtest of the NEUROPSI) neuropsychological tests as predictors. This predictive model correctly identifies 12/14 (86%) seronegative individuals diagnosed with suspicion of HAND (*S_e_* = 0.877, *S_p_* = 0.826, CCR = 0.833), and 31/36 (86.1%) individuals with HIV-1 infection meeting the IHDS criterion for HAND (*S_e_* = 0.861, *S_p_* = 0.917, CCR = 0.883). Overall, the model correctly classifies 43/50 individuals (86%; 31 with HIV-1 infection), leading to *S_e_* = 0.86, *S_p_* = 0.857, CCR = 0.858, and AUC = 0.859 (Figure 2b and Table 4). Thus, individuals with less than ten years of education have a 71% chance of HAND affection (47% of the total sample); this figure increases to 82% if less than 8.5 points are obtained in T9 (37% of the total sample). Besides, individuals with more than ten years of education, <44 points in T4, and <68 points in T11, have a 78% chance of HAND affection using the IHDS criterion (8% of the total sample). Unlike in the MMSE criterion (Figure 2a), both years of education and the individuals’ age are seemingly important predictors of HAND status when used by the IHDS criterion.

Finally, if both the MMSE and the IHDS operational criteria are used for screening to define HAND suspects, eight individuals in our sample (seven with HIV-1 Infection) met the diagnosis criteria (Table 5). We derived a three-level classification tree with three splitting nodes; these nodes correspond to the T4, the T16 (Score of the Motor Skills 2 subtest of the NEUROPSI), and the T21 tests (Figure 2c). Interestingly, our neuropsychological short protocol correctly identifies suspicion of HAND in 57/58 (98.3%) individuals with HIV-1 infection (*S_e_* = 0875, *S_p_* = 1, CCR = 0.983, AUC = 0.937) (Figure 2c and Table 5). Thus, individuals with <30 points in T4 and <1.5 points in T16 have a 58% chance of HAND (10% of the total sample); besides, this figure increases to 100% when more than 32 points are obtained in T21.

### 3.2. Specific Cut-Off Values for HAND Screening in This Caribbean Community

**Figure 3:**
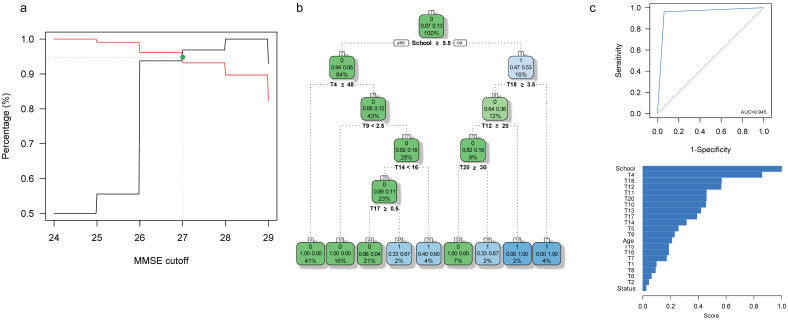
(**a**) Sensitivity (*S_e_*; black line) and specificity (*S_p_*; red line) as a function of the cut-off value for the MMSE in this Caribbean community. The optimal cut-off point is shown in green. (**b**) An ARPA-based predictive model for HAND screening when the MMSE optimal cut-off value is used to define HAND in all 120 assessed individuals. (**c**) ROC curve and variable importance for the derived model. Status: HIV-1 Infection status (0: control; 1: case). Other conventions as in Figure 1.

In our sample, the MMSE Score ranged from 23 to 29, and the IHDS score ranged from 3.5 to 12.5. Using the iterative process outlined in the methods section, we identified that MMSE < 27 (Figure 3 and Table 6) and IHDS < 10 (Figure 3 and Table 6) are the optimal cut-off values for screening suspicion of HAND in this Caribbean community.

When using an MMSE cut off value of <27, 16 individuals with suspicion of HAND are identified (14 with HIV-1 Infection; Table 6). Our predictive model for HAND status includes the T4, T9, T12 (Total Score in the Controlled Word Association Test [CWAT]), T14 (letter “M” Score of the CWAT), the T17 (Score of the Motor Skills 3 subtest of the NEUROPSI), T18 (Total Score in the Motor Skills subtests of the NEUROPSI), and T20 (Score of the State-Trait Anxiety Inventory [State]). This ARPA-based predictive model of HAND status yielded *S_e_* = 0.937, *S_p_* = 0.962, CCR = 0.958, and AUC = 0.949 for the whole sample, with similar results when only individuals with HIV-1 infection are considered (*S_e_* = 0.875, *S_p_* = 1, CCR = 0.983, AUC = 0.937) (Figure 3 and Table 6). In particular, individuals with less than 5.5 years of education have a 53% chance of being diagnosed with HAND (16% of the total sample; Figure 3); this figure increases to 100% when, in addition, individuals obtain either <3.5 points in T18 (4% of our sample) or less than 20 points in T12 (2% of our sample). On the other hand, individuals with <5.5 years of education, T18 ≥ 3.5, T12 ≥ 20, and T20 < 30 have a 67% chance of being diagnosed with HAND (2% of the sample). It is noteworthy that, in this model, years of education seems to be the most important variable when defining a HAND suspect.

**Figure 4:**
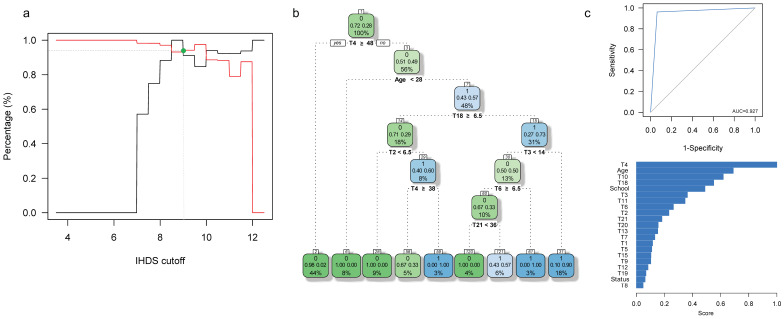
(**a**) Sensitivity (*S_e_*; black line) and specificity (*S_p_*; red line) as a function of the cut-off value for the IHDS in this Caribbean community. The optimal cut-off point is shown in green. (**b**) The ARPA-based predictive model for HAND when the IHDS optimal cut-off value is used to define HAND in all 120 assessed individuals. (**c**) ROC curve and variable importance for the derived model. Other conventions as in Figure 1.

At IHDS < 10, 34 individuals with suspected HAND are identified (26 with HIV infection; Table 6). Our predictive model includes age and the T2 (direct digits score of the Wechsler Memory Scale Digit Span subtest), T3 (Score of the Wechsler Intelligence Scale Vocabulary subtest), T4, T6 (Trial 5 of the Rey Auditory Verbal Learning Test), T18, and T21 tests as predictors of HAND status (Figure 4). This predictive model yields *S_e_* = 0.912, *S_p_* = 0.942, CCR = 0.933, and AUC = 0.927 in all 120 individuals, with similar performance measures for HIV-1 infected (*S_e_* = 0.923, *S_p_* = 0.882, CCR = 09.9, AUC = 0.903) and seronegative (*S_e_* = 0.875, *S_p_* = 0.981, CCR = 0.967, AUC = 0.928) individuals separately (Figure 4 and Table 6). Thus, individuals with <48 points in T4, and 28 years or older have a 57% chance of being classified as suspicion of HAND (48% of our sample). This figure increases to 73% when, in addition, individuals obtain less than 6.5 points in T18 (31% of our sample) and to 90% when individuals obtain more than 14 or more points in T3 (18% of our sample) (Figure 4).

## 4. Discussion

The detection and better understanding HAND accurately are critical for the clinical management of patients who suffer from this disorder [11]. In this study, we proposed a short neuropsychological protocol to detect HAND and evaluated its performance in individuals with asymptomatic HIV-1 Infection from an Afro-Colombian community in Barranquilla, Colombia. Our results show that our short neuropsychological protocol outperforms other instruments used for the same purpose in the clinical setting (Figure 1b and Table 4), such as the UCSD Performance-Based Skills Assessment (UPSA-B), which only has an accuracy of 71% when identifying HIV-1 infected individuals with neurocognitive impairment [62]. With the results obtained, we suggest that our short protocol could be a plausible evaluation tool for accurate HAND detection in clinical practice. Although our protocol is neither a screening test nor a full battery, it resembles other universally used standard neuropsychological tests that are sensitive in screening and monitoring for HAND [29], especially for mild HIV-related neurocognitive impairment [26]. It also allows for a more complete and time-effective evaluation in about 1 h, which makes it appealing in the clinical setting. Importantly, the ability of our short neuropsychological protocol for HAND screening in individuals with asymptomatic HIV-1 Infection highlights its importance to early diagnoses milder forms of HAND.

Highlighting the importance of some neuropsychological tests in our protocol is crucial in understanding the breakdown of such (Figure 1, Figure 2, Figure 3 and Figure 4). Namely, the Digit Symbol-Coding Subtest of the Wechsler Intelligence Scale (WAIS-III) [63], the Motor Skills Subtest of the Brief Neuropsychological Assessment in Spanish (NEUROPSI), the phonemic clues subtest of the Boston Naming Test, and the Controlled Word Association Test are designed to identify alterations in the speed of processing information, motor skills, and executive functions and are highly appropriate with the clinical description in HAND [64,65,66,67,68]. In addition to neuropsychological testing, the State-Trait Anxiety Inventory’s (STAI) ability to assess anxiety as part of the clinical features in HAND has previously been emphasized [69]. Furthermore, considering that age is a risk factor for HAND [70], it is noteworthy that age and years of education are essential variables to predict HAND in this population (Figure 1, Figure 2, Figure 3 and Figure 4). These variables may correspond to an early-stage profile of neurocognitive impairment (NCI) of the subcortical type, characterized by slower cognitive processes and reflected in attention, concentration, and executive function [6,71].

HAND diagnosis is not straightforward due to the existence of other clinical factors associated with HIV-1 infection [4,72,73] such as virus subtypes (clades) [74], high viral load, and low CD4 counts [75], HAND-associated risk factors [76,77], long-term exposure to cART [78], comorbidities [31,79,80], sociocultural/ethnic backgrounds [15,16,17,64] and genetic factors [81,82]. Interestingly, compared to using the proposed neuropsychological protocol, including CD4 counts as a predictor does not significantly improve the accuracy of the ARPA-based predictive model of HAND in patients with asymptomatic HIV-1 infection (Figure S1) Therefore, a straightforward neuropsychological evaluation for HAND is crucial to the diagnosis [24], and it is important to strengthen and unify methods of screening to assess milder to severe manifestations of HAND efficiently. It also allows for a more effective follow up of patients using evaluations emphasizing clinical variables associated with this neurocognitive disorder [64,83]. These new processes should be considered as part of the integrative care of individuals with HIV-1 Infection and therefore included in the routine clinical HIV management [84].

We found that our ARPA-based HAND predictive model, involving the MMSE criterion (Figure 1a), yielded outstanding specificity and correct classification rates, but low-to-average specificity and AUC (Table 4). Thus, we advise against using this criterion to define HAND in individuals with asymptomatic HIV-1 Infection. Low performance in the MMSE might be indicative of a possible neurocognitive impairment (NCI) of cortical origin [85], which is present in more advanced HAND states [86], and it is also associated with more advanced stages of the Infection [86,87]. It could partially explain why our clinical protocol detects more HAND cases among individuals with asymptomatic HIV-1 Infection. Furthermore, it is highly likely that one would not expect a more significant neurocognitive compromise because of the asymptomatic nature of the Infection. Frontal white matter compromise has been demonstrated in ANI, while a more widespread subcortical atrophy has been found in MND [88]. However, individuals with HIV-1 Infection, where HAND was detected, could have more severe clinical characteristics that potentially explain the neurocognitive compromise. These results may also be a consequence of the low sensitivity of the MMSE to detect HAND, especially in the early stages [3,89,90,91].

The IHDS is a useful tool to screen HAND [3,10]; it has a higher sensitivity to detect HAD [8,18], but performs poorly to detect milder forms of HAND [89]. This scale should be preferred in situations where sensitivity is more important at the expense of its loss of specificity [25], and as a screening tool in scenarios with limited resources [23]. In our sample, the IHDS correctly identifies what seems to be milder forms of HAND because there is no compromise of daily living activities [7] (Figure 1b, Figure 2b, and Table 4). Thus, our findings support the use of the IHDS to detect milder forms of HAND [63].

We also found that when using the IHDS-based operational criterion as a screening tool for HAND, most of the diagnoses were assigned to individuals with HIV-1 Infection (Figure 2b and Table 5). It implies a higher sensitivity of this criterion to identify the HAND of subcortical origin, which corresponds to the early stages [6]. Although the IHDS was specifically designed to identify this NCI profile in individuals with HIV-1 Infection [23], our IHDS-based screening tool (Figure 2b) also detects 14 seronegative individuals who, in principle, are not affected by NCI (Table 5). This result may be a consequence of the previously reported lack of specificity of the IHDS [25], and could also be explained by the sociodemographic characteristics of this Caribbean community [23], associated with elements of the acculturation which can affect neuropsychological functioning [92].

Although the MMSE and IHDS screening tools were designed to assess the brief cognitive state, the performance of this combined HAND screening tool outperforms that of either the MMSE (Figure 2a and Table 5) or IHDS (Figure 2b and Table 5) criterion. One possible explanation is that the MMSE + IHDS operational criterion is effectively assessing different cognitive functions. It means that one test does not substitute the other, and their combination improves HAND diagnostic accuracy [5]. Thus, we strongly advocate for a multidisciplinary approach to HAND [93] where pathophysiological biomarkers [94], neurological and psychiatric differential diagnosis [95], and other confounding variables [7], together with a neuropsychological assessment, comprise the ideal set of tools for the diagnosis of HAND.

Most research studies on HAND and its diagnosis have been conducted in North America, Europe, and Africa, with very few studies in Latin America. To the best of our knowledge, there are no available neuropsychological protocols for HAND detection in Colombia, let alone communities belonging to this geographical area (with specific patterns of ancestry and cultural background) [96]. Following the recommendations of experts [18,97], and considering the relevance of an early HAND diagnosis in individuals with HIV-1 Infection, one of the strengths of this study is the proposal of a short protocol that could be applied in approximately 1 h, is comprised by standardized tests widely used in different countries around the world, and has the potential to detect HAND from the asymptomatic stage of the HIV-1 infection. Thus, the ARPA-based predictive models of HAND are presumably closer to the diagnosis or screening of a milder form of HAND. Our results suggest that all derived models based on the neuropsychological battery previously reported (Table 2 and Supplementary Material) could be a suitable HAND detection tool in the clinical practice and may help in the early detection and follow-up of individuals with asymptomatic HIV-1 Infection who are at risk of developing HAND [6,70,81,85]. A potential disadvantage of our approach is that it has not yet been tested to detect HAND in more advanced stages of the infection. Hence, future additional studies are needed in this direction.

Despite our encouraging results, some limitations are to be acknowledged. First, we only recruited individuals with asymptomatic HIV-1 Infection, which impeded us from testing our ARPA-based predictive models for HAND in individuals with symptomatic HIV or AIDS. A second limitation is the sample size. Although we previously showed that a sample of 60 seronegative controls and 60 individuals with HIV-1 Infection has enough power to detect differences between the HIV-infected and control groups across the set of 21 neuropsychological tests administered to all subjects [39], this may not be the case for predictive models. A third limitation is the non-inclusion of other psychological [98], sociocultural/ethnic, or genetic factors that could potentially influence the prediction of HAND in our sample [15,16,17,81,82,99]. Despite these limitations, the clinical variables included in our neuropsychological protocol predict HAND status, with high accuracy, in this community (Figure 1 and Figure 4). As part of an integrative approach, we encourage the use of our short protocol in combination with other methods to detect HAND.

Future similar studies for this topic, especially those of longitudinal nature, should be conducted in Colombia and Latin America. Studies covering all stages of the HIV-1 Infection that include other clinical, medical, virologic, and psychosocial variables associated with HAND, in addition to the neuropsychological evaluation protocol and combination antiretroviral therapy (cART) being used, are needed. As cART are known to contribute to HAND due to the toxicity of these drugs, studies assessing how cART delineates HAND risk and are yet to be explored. Moreover, the identification of biomarkers underpinning HAND susceptibility [100] may be crucial for a better understanding of HAND, and will allow for more accurate diagnosis and follow-ups. This will converge in establishing clinical guidelines for HAND in the developing world [101].

## 5. Conclusions

The proposed HAND short neuropsychological protocol showed an outstanding performance to detect suspicion of HAND in individuals with asymptomatic HIV-1 Infection from an African-admixture community in Barranquilla, Colombia. By using three different operational criteria and developing community-specific cut-off values, we were able to derive normalized data and highly improve the prediction of HAND diagnosis in this population. This short neuropsychological protocol may help to increase the detection accuracy of milder forms of HAND in the clinical setting, and facilitate monitoring neuropsychological functioning, patients’ follow-up, and treatment decision making.

## Figures and Tables

**Table 1:** Demographic characteristics of participants.

Variable	Category	All Individuals (*n* = 120)	Patients with Asymptomatic HIV-1 (*n* = 60)	Control Group (*n* = 60)	Statistic	*p*
		Mean ± S.D.	Mean ± S.D.	Mean ± S.D.	*t* (df)	
Age (years)		36.07 ± 10.98	38.60 ± 9.48	33.53 ± 11.84	2.59 (118)	**0.011**
Years of education		9.02 ± 2.65	8.07 ± 2.85	9.97 ± 2.07	4.18 (118)	**<0.0001**
		***n* (%)**	***n* (%)**	***n* (%)**	$\chi^{2}$ **(*df*)**	
Gender	Female	74 (61.7)	32 (53.3)	42 (70)	3.52 (1)	0.060
	Male	46 (38.3)	28 (46.7)	18 (30)		
Sexual orientation	Heterosexual	111 (92.5)	53 (88.3)	58 (96.7)	4.42 (2)	0.109
	Homosexual	5 (4.2)	3 (5)	2 (3.3)		
	Bisexual	4 (3.3)	4 (6.7)	0 (0)		
Hand preference	Left	6 (5)	0 (0)	6 (10)	6.32 (1)	**0.012**
	Right	114 (95)	60 (100)	54 (90)		

Results significant at 5% are shown in bold. *df* = degrees of freedom; SD = Standard deviation. Note that HIV-1 infected and seronegative control groups differed in age and years of education.

**Table 3:** Number of individuals with HAND based on the short screening protocol following Frascati 1SD criterion, along with the ARPA-based predicted HAND diagnosis by HIV-1 infection status. Here, age, sex, years of education, and tasks comprising our short screening protocol were included as predictors.

Asymptomatic HIV-1 Infection	ARPA-Based Predicted HAND	HAND Diagnosis		Total
		No	Yes	
No	No	56	0	56
	Yes	4	0	4
Yes	No	0	5	5
	Yes	0	55	55

**Table 4:** Performance measures when different instruments are used in the ARPA-based predictive model of HAND. Overall, the proposed battery performs better than other instruments also evaluated. Here, *a* is the number of individuals with HAND that are correctly classified, *b* is the number of HAND individuals classified as controls, *c* corresponds to the number of control individuals classified as HAND, and *d* to the number of control individuals correctly classified. *S*_e_ = sensitivity; *S*_p_ = specificity; PPV = positive predictive value; NPV = negative predictive value; FDR = false discovery rate; FPR = false positives rate; CCR = Correct classification rate (Accuracy); AUC = area under the ROC curve (Figure 1b). See Supplementary Table S1 for expressions to calculate these performance measures form *a*, *b*, *c,* and *d*.

Instrument	*a*	*b*	*c*	*d*	*S*_e_	*S*_p_	PPV	NPV	FDR	FPR	CCR	Lift	AUC
Short Protocol	55	4	5	56	0.917	0.933	0.932	0.918	0.068	0.067	0.925	1.864	0.925
MMSE	50	11	10	49	0.833	0.817	0.820	0.831	0.180	0.183	0.825	1.639	0.825
IHDS	53	9	7	51	0.883	0.850	0.855	0.879	0.145	0.150	0.867	1.710	0.867
MMSE + IHDS	53	5	7	55	0.883	0.917	0.914	0.887	0.086	0.083	0.900	1.828	0.900

**Table 5:** Number of individuals with HAND and ARPA-based predicted HAND diagnosis by HIV-1 infection status. HAND diagnosis was defined according to three different criteria. For each criterion, age, sex, years of education, and tasks comprising our short screening protocol were used as predictors in the ARPA-based model.

**Criterion 1: MMSE in (10,25]**				
**Asymptomatic HIV-1 Infection**	**Predicted HAND**	**HAND Diagnosis**		**Total**
		**No**	**Yes**	
No	No	60	0	60
	Yes	0	0	0
Yes	No	50	4	54
	Yes	1	5	6
**Criterion 2: IHDS < 11**				
No	No	38	2	40
	Yes	8	12	20
Yes	No	22	5	27
	Yes	2	31	33
**Criterion 3: MMSE in (10,25] and IHDS < 11**				
No	No	60	0	60
	Yes	0	0	0
Yes	No	52	1	53
	Yes	0	7	7

**Table 6:** Number of individuals with HAND and ARPA-based predicted HAND diagnosis by HIV-1 infection status using the derived community-specific cut-off values for the MMSE and IHDS. HAND diagnosis was defined according to the community-specific cutoff values. Age, sex, years of education, and the neuropsychological tasks comprising our short screening protocol were used as predictors in the ARPA-based model.

MMSE < 27				
Asymptomatic HIV-1 Infection	Predicted HAND	HAND Diagnosis		Total
		No	Yes	
No	No	57	0	57
	Yes	1	2	3
Yes	No	43	1	44
	Yes	3	13	16
**IHDS < 10**				
No	No	51	1	52
	Yes	1	7	8
Yes	No	30	2	32
	Yes	4	24	28

## Supplementary Materials

The following are available online at https://www.mdpi.com/article/10.3390/brainsci11081037/s1, Figure S1: Results of the ARPA-based predictive model for HAND screening using the (a) MMSE, (b) IHDS and (c) MMSE+IHDS criteria; Table S1: Possible results when comparing real and predicted HAND; Table S2: Expressions for calculating the performance measures used to quantitatively compare the performance of the different instruments used to predict HAND using our ARPA-based predictive model.
