# Supplementary material for: Utility of a Short Neuropsychological Protocol for Detecting HIV-Associated Neurocognitive Disorders in Patients with Asymptomatic HIV-1 Infection
Source: Brain Sci. 2021 Aug 4;11(8):1037. doi: 10.3390/brainsci11081037 (PMC8394998; doi:10.3390/brainsci11081037)
Supplement: Supplementary file 1 [file brainsci-11-01037-s001.zip › brainsci-1281250-supplementary.pdf]

brain sciences

MDPI

Supplementary Material

Utility of a Short Neuropsychological Protocol for Detecting HIV-Associated Neurocognitive Disorders in Patients with Asymptomatic HIV-1 Infection

Martha Martinez-Banfi [1,*], Jorge I. Vélez [2], Moisés Mebarak Chams [2], Mauricio Arcos-Holzinger [3], Johan Acosta-López [1], Ricardo García [4], M. Victoria Perea [4], Mauricio Arcos-Burgos [3] and Valentina Ladera [4]

1 Universidad Simón Bolívar, Facultad de Ciencias Jurídicas y Sociales, Barranquilla 080005, Colombia; jacosta@unisimonbolivar.edu.co
2 Universidad del Norte, Barranquilla 081007, Colombia; jvelezv@uninorte.edu.co (J.I.V.); mmebarak@uninorte.edu.co (M.M.C.)
3 Grupo de Investigación en Psiquiatría (GIPSI), Departamento de Psiquiatría, Instituto de Investigaciones Médicas, Facultad de Medicina, Universidad de Antioquia, Medellín 050010, Colombia; oscararcos98@gmail.com (M.A.-H.); mauricio.arcos@udea.edu.co (M.A.-B.)
4 Facultad de Psicología, Universidad de Salamanca 37008, España; rigar@usal.es (R.G.); vperea@usal.es (M.V.P.); ladera@usal.es (V.L.)
* Correspondence: mmartinez108@unisimonbolivar.edu.co

Variables and instruments

The proposed neuropsychological evaluation protocol used in this research was constructed from the review of similar studies in the HIV-infected population [1–4]. The instruments used are described as follows:

Mini-Mental State Examination[5]

The Mini-Mental State Examination is a short, easy-to-administer screening test that evaluates general cognitive functioning. It consists of a 30-item questionnaire that evaluates temporal and spatial orientation, working memory, attention and calculation, delayed recall, and language and construction.

International HIV dementia scale (IHDS)[6]

This is a brief screening instrument designed to evaluate the existence of a possible subcortical HAD. The IHDS consists of three subtests namely timed finger tapping, timed alternating hand sequence test, and recall of four items 2 minutes after administration. The maximum possible score is 12 points. Individuals with an IHDS score of ≤ 10 should be subsequently evaluated for possible dementia.

Weschler Intelligence Scale [7]

The vocabulary subtests and number-coding key were used from the Weschler Intelligence Scale. As its name says, the former evaluates vocabulary, and the subject's task is to orally define a series of words that the examiner reads aloud, one by one, from vocabulary cards. The latter evaluates the speed of information processing and consists of the subject copying a series of symbols that are each matched to a number, with a maximum time of 120 seconds.

Weschler Memory Scale Digits Subtest [8]

This test consists of two parts that are independently applied (in forward and reverse order), and its objective is to measure the subject’s attention span (Lezak, Howieson and Loring, 2004).

Rey's Auditory-Verbal Learning Test [9, 10]

The version of the test used in this study (Schmidt, 1996) is an instrument consisting of a list of 15 words, which are read to the patient at a rate of one word per second. The patient is asked to recall the words, regardless of the order. Subsequently, a learning curve is established by presenting the word list five times (5 trials). Afterwards, a second list of words that is different from the first list is read to the patient, and the patient is asked to repeat the words they recall from the second list. The patient is then again asked to recall the words from the initial list (trial 6). Finally, the patient is asked to recall the original list again 20 minutes after the completion of the administration of the test (trial 7).

Rey's Complex Figures Test [11]

This test consists of two phases: a copying phase and a memory reproduction phase. In the copying phase, the subject's visual perceptual capacity, praxical-constructive abilities and visuo-spatial analysis capacity are assessed. In the second phase, the memory reproduction phase, after a short pause not to exceed three minutes, the subject is asked to reproduce the copied figure from memory (Rey, 2003). In this second phase, the subject's visuoconstructive memory capacity can be observed.

Boston Vocabulary Test [12]

The Boston Vocabulary Test was designed to measure the nominative function of language and is part of the Boston Aphasia Exam. There are several versions, but the original version, used in this study, consists of 60 monochromatic drawings arranged on one sheet each.

Controlled Word Association Test [13]

The version used in this study is described by Spreen and Strauss [13]. It evaluates phonological verbal fluency using a word search task and is sensitive to left frontal lobe dysfunction.

STROOP Test of Colors and Words[14]

This instrument measures response inhibition. The test consists of three pages containing 100 elements. The first page consists of the words "red", "green", and "blue" randomly ordered and printed in black ink; the second page is consists of the element "XXXX" printed in blue, green and red ink; and the third page consists of the words on the first page printed in the colors of the second page, mixed item-by-item.

The Trail Making Test (TMT), from the Spanish Stroke Test [15]

The TMT consists of two parts: A and B. Part A consists of joining (in order) a group of numbers enclosed in circles as fast as possible, and part B consists of joining (in order) a group of numbers and letters while also alternating the numbers and letters.

Brief Neuropsychological Evaluation in Spanish [16]

The language comprehension subtest from the Brief Neuropsychological Evaluation in Spanish was used and is designed to specifically evaluate language comprehension. In addition, the motor functions subtest, which consists of repeating three different series of hand movements that were previously demonstrated by the examiner, was also used.

Table S1. Possible results when comparing real and predicted HAND. Here, *a* is the number of individuals with HAND that are correctly classified, *b* is the number of HAND individuals classified as controls, *c* corresponds to the number of control individuals classified as HAND, and *d* to the number of control individuals correctly classified.

**Phenotype**	**Prediction**	
	**HAND**	**Control**
HAND	*a*	*b*
Control	*c*	*d*

Table S2. Expressions for calculating the performance measures used to quantitatively compare the performance of the different instruments used to predict HAND using our ARPA-based predictive model. See Table S1 for more information.

**Measure**	**Expression**
Sensitivity (S_e)	a / (a+c)
Specificity (S_p)	d / (c+d)
Positive predictive value (PPV)	a / (a+b)
Negative predictive value (NPV)	d / (c+d)
False Discovery Rate (FDR)	1 - *PPV*
False Negatives Rate (FNR)	1 - *NPV*
Lift	$a\ n$ / {(a + b)(a + c)}
Classification rate (Accuracy)	(a+d) / n

n = a + b + c + d.

Use of CD4 counts to predict HAND

CD4 counts and viral load are predictors of HAND status in general. In our case, CD4 counts were only available for HIV-1 infected individuals. Thus, we fitted an ARPA-based predictive model as described in the Methods section with this set of individuals using demographic variables and CD4 counts as predictors. HAND diagnosis was assessed using the MMSE, IHDS and MMSE+IHDS criterion. Out of the 60 individuals with HIV-1 Infection, 9, 36 and 8 were diagnosed with HAND according to the MMSE, IHDS and MMSE+IHDS criterion (Table 4 of the main manuscript).

Using CD4 counts and demographic variables as predictors of HAND based on the MMSE criterion led to S_e, S_p and CCR values of 0.445, 0.98 and 0.9, respectively, with an AUC of 0.712. When the IHDS criterion was used, the ARPA-based predictive model led to S_e, S_p, CCR and AUC values of 0.889, 0.833, 0.867 and 0.861, respectively. Finally, when the MMSE+IHDS criteria was used, the predictive model led to S_e, S_p, CCR and AUC values of 0.5, 0.98, 0.917 and 0.74, respectively. Although these resulting performance measures are acceptable, our proposed brief protocol for HAND screening outperforms the use of CD4 counts to predict HAND status (see Table 3 and subsection 3.1.2 of the main manuscript for more details). Figure 1S depicts the tree-based model and ROC curves.

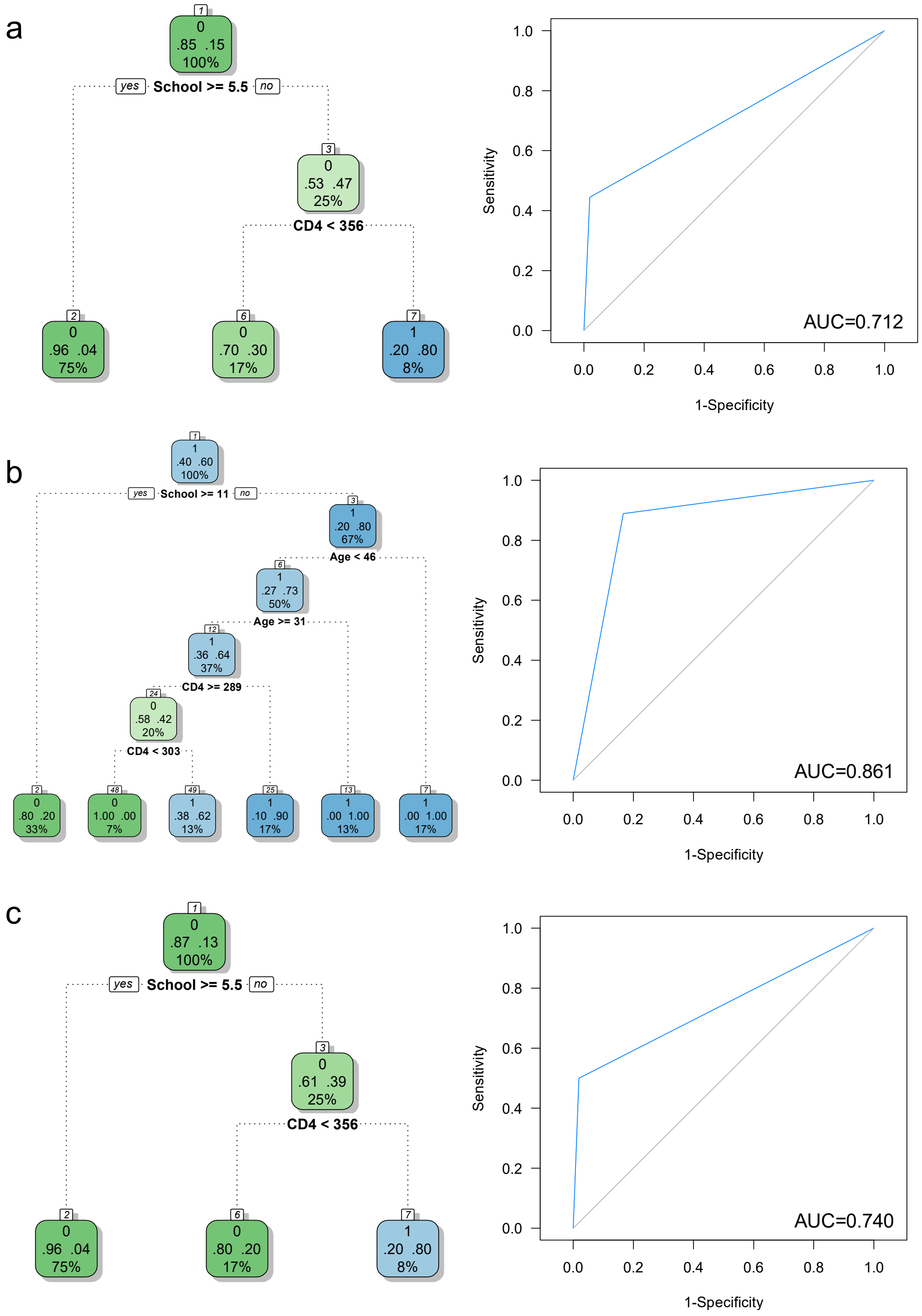

Figure S1. Results of the ARPA-based predictive model for HAND screening using the (a) MMSE, (b) IHDS and (c) MMSE+IHDS criteria. Here, CD4 counts and demographic variables are used as predictors. Only individuals with HIV-1 Infection are included. Other convections as in Figure 1 in the main manuscript.

References.

[1] Ardila-Ardila A, Goodkin K, Concha-Bartolini M, et al. [HUMANS: a neuropsychological battery for evaluating HIV 1 infected patients]. *Rev Neurol* 2003; 36: 756–62.
[2] Ayuso Mateos JL. *Trastornos neuropsiquiátricos en el SIDA*. McGraw-Hill Interamericana, https://www.casadel-libro.com/libro-trastornos-neuropsiquiatrico-del-sida/9788448601768/562347 (1997, accessed 6 April 2018).
[3] Butters N, Grant I, Haxby J, et al. Assessment of AIDS-related cognitive changes: recommendations of the NIMH Workshop on Neuropsychological Assessment Approaches. *J Clin Exp Neuropsychol* 1990; 12: 963–78.
[4] Maj, M., Starace, F., y Sartourius N. Neuropsychiatric aspects of HIV-1 infection: data collection instrument for a WHO cross-cultural study. *Organ* 1991; 69: 243–245.
[5] Folstein, Marshal F., Susan E. Folstein and PRM. 'Mini-mental state'. A practical method for grading the cognitive state of patients for the clinician. *J Psychiatr Res* 1975; 12: 189–198.
[6] Sacktor NC, Wong M, Nakasujja N, et al. The International HIV Dementia Scale: a new rapid screening test for HIV dementia. *AIDS* 2005; 19: 1367–74.
[7] Weschler D. *Manual de aplicación y corrección de la escala de inteligencia de Weschler (WAIS-III)*. Madrid, 2001.
[8] Weschler D. *Escala de memoria de Weschler-III*. Madrid, 2004.
[9] Rey, A. (1964) L'examen clinique en psychologie (The Clinical Psychological Examination). Presse Universitaires de France, Paris. - References - Scientific Research Publishing, https://www.scirp.org/(S(vtj3fa45qm1ean45vvffcz55))/reference/referencespapers.aspx?referenceid=1656248 (ac-cessed 2 June 2021).
[10] Schmidt M. *Rey Auditory and Verbal Learning Test: A handbook*. Los Angeles, CA, 1996.
[11] Rey A. *Test de copia y de reproducción de memoria de figuras geométricas complejas. [Test of copying and reproduction of memory of complex geometric figures.]*. 8th ed. Madrid, 2003.
[12] Kaplan, EF, Goddglass, H. and Weintraub S. *Boston Naming Test*. 2nd ed. Buenos Aires, 1983.
[13] Spreen, O. and Strauss E. *A compendium of neuropsychological tests*. 2ª ed. New York, 1998.
[14] Golden CJ. *Test de colores y palabras STROOP. [STROOP Test of colors and words]*. (4th ed.). Madrid, 2005.
[15] Lezak M., Howieson, D. and Loring D. *Neuropsychological Assessment*. 2nd ed. Estados Unidos, 1983.
[16] Otrosky-Solis, F., Ardila, A. and Rosselli M. *Evaluación Neuropsicológica breve en español (Neuropsi). [Brief Neuropsy-chological evaluation in Spanish (Neuropsi).]*. Mexico, 1999.
